# How temperature reshapes interactions in mixed cultures of mycotoxigenic fungi

**DOI:** 10.3389/fmicb.2026.1903639

**Published:** 2026-08-24

**Authors:** Darina Balková, Marco Camardo Leggieri, Paola Battilani

**Affiliations:** Department of Sustainable Crop Production, Università Cattolica del Sacro Cuore, Piacenza, Italy

**Keywords:** *Aspergillus flavus*, climate change, co-occurrence, *Fusarium graminearum*, *Fusarium verticillioides*, growth kinetics, qPCR

## Abstract

Climate change is shifting the distribution of mycotoxigenic fungi, which may alter patterns of co-occurrence in staple crops. Yet, quantitative, reproducible *in vitro* approaches to characterise multispecies growth and interaction outcomes under environmentally relevant conditions remain limited. Here, we developed a small-scale workflow to quantify temperature-dependent interaction outcomes among three major cereal-associated fungi, *Aspergillus flavus, Fusarium verticillioides*, and *F. graminearum*. High-frequency optical density time series were collected in 96-well liquid cultures across 10 °C–45 °C. Optical density trajectories were summarised with three-parameter logistic fits used as empirical descriptors of culture-level dynamics (carrying capacity, growth rate, and inflexion point). Species-specific quantitative PCR was then used to quantify endpoint DNA-based species contributions in mixed cultures. Monoculture responses were consistent with established thermal preferences. In co-cultures, optical density-derived interaction patterns indicated a shift in mixed-culture optical performance towards warmer conditions. Quantitative PCR endpoint composition supported an increasing contribution of *A. flavus* at elevated temperatures, with reduced contributions of both *Fusarium* species. These results show that temperature reshapes interaction outcomes through changes in culture-level growth kinetics and endpoint community composition. The workflow provides data suitable for predictive modelling of climate-driven mycotoxin risk and supports interpretation of fungal interaction outcomes in warming agroecosystems.

## 1 Introduction

Climate change is one of the most complex global challenges, impacting ecosystems, agriculture, and food safety worldwide. Shifts in weather patterns are reshaping natural habitats and species interactions (Calvin et al., 2023), increasing stress on plant vitality and intensifying disease pressures across agroecosystems (Singh et al., 2023). Global warming has been associated with a higher proportion of soil-borne plant pathogens including fungi (Delgado-Baquerizo et al., 2020), whose growth, reproduction, and secondary metabolism are closely linked to temperature and water availability (Magan et al., 2011; Elad and Pertot, 2014). These changes contribute to increasing prevalence and geographical expansion of toxigenic fungal species (Casu et al., 2024), which are able to contaminate agricultural commodities at multiple stages of the production chain with toxic secondary metabolites (mycotoxins) [(European Food Safety Authority (EFSA) et al., 2020)].

Among the mycotoxigenic fungi affecting cereals, *Aspergillus flavus, Fusarium verticillioides*, and *F. graminearum* are important due to their association with major regulated mycotoxins: *A. flavus* produces aflatoxins (AFs), *F. verticillioides* produces fumonisins (FBs), and *F. graminearum* produces deoxynivalenol (DON) and zearalenone (ZEN) (Schatzmayr and Streit, 2013; Alshannaq and Yu, 2017). These species are associated with maize and other cereal crops and differ in their ecological and thermal requirements, making their potential co-occurrence relevant under changing climatic conditions. Multi-year surveys indicate that contamination is common, with associations to weather variability (Gruber-Dorninger et al., 2019; Palumbo et al., 2020). In Europe, warmer conditions have been associated with aflatoxin risk in maize (Battilani et al., 2016; Bereziartua et al., 2025). Experimental studies have demonstrated that environmental factors such as temperature, water activity, and CO_2_ influence fungal growth and physiological responses, including AFs biosynthesis (Medina et al., 2015, 2017). Risk projections indicate a progressive northward shift of risk zones for *Aspergillus* and *Fusarium* species under future climate scenarios (Van der Fels-Klerx et al., 2013, 2016), consistent with poleward pathogen movement (Bebber et al., 2013).

Increasing evidence shows that *A. flavus, F. verticillioides*, and *F. graminearum* frequently co-occur within the same host or environment (Gruber-Dorninger et al., 2019; Penagos-Tabares et al., 2025; Hao et al., 2023). Co-occurrence of *A. flavus* and *F. verticillioides* has been reported in maize in semi-arid and warming temperate regions (Lanubile et al., 2021; Chen et al., 2023), and field experiments indicate that simultaneous presence of *A. flavus, F. verticillioides*, and *F. graminearum* can influence fungal incidence and toxin profiles (Giorni et al., 2019). Generally, interactions among species can modify growth dynamics, competitiveness, and relative abundance in ways not predictable from monocultures (Camardo Leggieri et al., 2019; Chuaysrinule et al., 2023), and may suppress or enhance mycotoxin production depending on environmental conditions, niche overlap and substrate competition (Leggieri et al., 2020). This can contribute to multi-mycotoxin contamination, which complicates risk assessment and management, since regulatory frameworks typically address single toxins, while combined exposures are common (Palumbo et al., 2020; Gruber-Dorninger et al., 2025).

Despite well-documented co-occurrence cases, quantitative, reproducible *in vitro* approaches for assessing how temperature shapes multispecies growth remain limited. Traditional plate assays capture colony diameter, but are time and labour intensive, limited in number of replicates, and provide only semi-quantitative data. This complicates extraction of reproducible growth parameters and identification of species-specific contributions in mixed systems. Here, we developed a reproducible small-scale workflow to quantify how temperature shapes mixed-culture behaviour in three major mycotoxigenic fungi. The approach combines 96-well liquid co-cultures and automated high-frequency optical density monitoring across a controlled temperature gradient (10 °C–45 °C), with logistic modelling used as an empirical summary of culture-level growth kinetics, and species-specific quantitative PCR used to resolve endpoint DNA-based species composition in mixed cultures. Using monocultures, dual cultures, and a three-species culture, our aims were to: (1) characterise temperature-dependent growth patterns across culture types; (2) compare culture-level optical responses between single- and mixed-species conditions; (3) quantify endpoint species contributions within mixed cultures; and (4) derive quantitative descriptors of interaction outcomes across experimental conditions.

## 2 Materials and methods

### 2.1 Fungal strains and inoculum preparation

The fungal isolates *A. flavus* (ITEM 8096), originally isolated from maize grown in Piedmont (Italy) and capable of aflatoxin B_1_ production, *F. verticillioides* (ITEM 10027), isolated from maize grown in Emilia-Romagna (Italy) and capable of fumonisin production, and *F. graminearum* (ITEM 646), isolated from durum wheat kernels grown in Basilicata (Italy) and capable of deoxynivalenol production, were used for inoculum preparation. These isolates are maintained in the official fungal collection of the Institute of Sciences of Food Production of the National Research Council (ISPA-CNR; Bari, Italy) and were selected as representative cereal-associated strains previously characterised in ecophysiological studies (Lanubile et al., 2021; Giorni et al., 2009; Lazzaro et al., 2012). Strains were individually inoculated on potato dextrose agar (PDA; Biolife, Milano, Italy) plates (9 cm diameter) and incubated at 25 °C (12 h light/12 h dark photoperiod) for 7 days. After incubation, plates were washed with sterile distilled water (10 mL), filtered through a textile cloth, and each suspension was adjusted to 10^6^ spores mL^−1^ using a hemocytometer.

### 2.2 Media preparation and microplate co-culture assay

Potato broth (PB) was prepared from 400 g washed and diced potatoes autoclaved in 1,200 mL distilled water and filtered through a metal mesh. For 1 L medium, 330 mL PB was diluted to 1,000 mL with distilled water, supplemented with 20 g glucose, autoclaved, and stored at 4 °C until use. For the co-culture microplate assay, 100 μl liquid medium was dispensed into each well of a sterile 96-well round-bottom microplate with lid (SARSTEDT AG & CO, Germany) under laminar flow. Wells were inoculated with 100 μl conidial suspension at 10^6^ spores mL^−1^ (or sterile distilled water for negative controls). Each row represented one treatment (12 wells per treatment), including monocultures, dual-species combinations, the three-species combination, and a negative control (100 μl medium + 100 μl sterile distilled water). For co-inoculations, suspensions were mixed in equal proportions (50:50 for dual cultures; 33:33:33 for the three-species culture), maintaining equal total inoculum volume and spore concentration across treatments. The complete microplate experiment was conducted twice as two independent experimental runs under identical conditions. Both runs were analysed separately and showed comparable growth patterns. The results presented in this manuscript correspond to the second experiment. The microplate layout is shown in Supplementary material.

### 2.3 Spectrophotometric growth monitoring

Fungal growth was monitored using a Microplate Absorbance Reader 800 TS (BioTek Instruments, Agilent Technologies, USA). Optical density (OD) was measured at 405, 550, and 620 nm (Langvad, 1999). Preliminary trials showed no statistically significant differences among wavelengths; therefore, OD at 405 nm was used for all subsequent analyses, consistent with previous studies (Slowik et al., 2023). Microplates were incubated with lids featuring individual well rims to reduce evaporation and limit cross-well contamination. Readings were recorded automatically every 11 mins after 10 s linear shaking over 168 h at eight temperatures (10 °C, 15 °C, 20 °C, 25 °C, 30 °C, 35 °C, 40 °C, and 45 °C). Each treatment–temperature combination was represented by *n* = 12 wells per plate. Protocol and plate layout were programmed in the instrument software before acquisition.

### 2.4 Growth curve processing and logistic modelling

Raw OD values were imported and processed in Python 3.11 (Python Software Foundation, Wilmington, DE, USA) using pandas, NumPy, and SciPy. Baseline-corrected net growth (ΔOD) was calculated for each well by subtracting the first time-point value of that well. Means and standard deviations were then calculated across replicates for each treatment and time point.

Growth dynamics were described using a three-parameter logistic model:


$$\begin{array}{l} \text{ΔOD}\left(t\right)=\frac{K}{1+\exp\left[-r\left(t-t_{0}\right)\right]}, \end{array}$$

where *K* is carrying capacity (maximum ΔOD), *r* is growth rate, and *t*_0_ is the inflexion point (time of maximum growth rate), as previously applied to microbial growth data (Sprouffske and Wagner, 2016; Simpson et al., 2022; Lo Grasso et al., 2023). The logistic model was used as an empirical descriptor of curve shape to enable comparison across treatments. A schematic representation is provided in Supplementary Figure 2. Models were fitted by nonlinear least-squares regression using scipy.optimize.curve_fit. Each fit was validated using classical *R*^2^, the empirical fit-quality score *R*^2*^, and NRMSE, as detailed in the Statistical analysis subsection. Model acceptance required ΔOD_max_ ≥ 0.10 and *R*^2*^ ≥ 0.95. Fit-quality metrics for all 56 treatment–temperature combinations are reported in Supplementary Figure 3. Fits that did not meet these criteria were excluded from interpretation of *K*, *r*, and *t*_0_.

### 2.5 Quantification of interaction effects

Interaction effects were quantified from maximum baseline-corrected ΔOD (ΔOD_max_) at 168 h. Values were calculated per well and summarised as mean ± SD per treatment. Because OD measurements in filamentous fungi are influenced by species-specific mycelial morphology and optical scattering (Meletiadis et al., 2001), OD-derived metrics in mixed cultures were interpreted as culture-level optical descriptors.

For each focal species *i*, a normalised performance index (NPI) at temperature *T* was calculated on a 0–1 scale using the monoculture ΔOD_max_ range across temperatures:


$$\begin{array}{l} {\text{NPI}}_{i,T}=\frac{G_{i,T}-G_{i,\min}}{G_{i,\max}-G_{i,\min}}, \end{array}$$

where *G*_*i, T*_ is mean ΔOD_max_ at temperature *T*, and *G*_*i*, min_ and *G*_*i*, max_ are the minimum and maximum monoculture ΔOD_max_ values of species *i* across the full temperature range. In mixed cultures, *G*_*i, T*_ denotes the total optical signal of the co-culture containing species *i* (not a species-specific biomass estimate). Co-culture values were normalised using the same monoculture-derived scale, allowing values >1 or < 0.

Deviations from monoculture reference were expressed as:


$$\begin{array}{l} \text{Δ}{\text{NPI}}_{i,T}={\text{NPI}}_{i,T}^{\text{co-culture}}-{\text{NPI}}_{i,T}^{\text{monoculture}}, \end{array}$$

where positive values indicate enhanced co-culture optical performance and negative values indicate reduced co-culture optical performance relative to monoculture at the same temperature. Similar species-focused normalisation approaches have been used in other quantitative studies using microplate measurements (McBirney et al., 2016; Slowik et al., 2023).

### 2.6 Genomic DNA extraction and qPCR analysis

Fungal genomic DNA (gDNA) was extracted at incubation endpoint (168 h) using the Quick-DNA Fungal/Bacterial Miniprep Kit (Zymo Research), following the manufacturer's protocol. Four wells per treatment were pooled per sample to obtain sufficient material, yielding *n* = 3 biological replicates. To assess positional/edge effects in microplates, pooled biomass was weighed before DNA extraction; no systematic biomass differences were observed. To remove residual medium, mycelia were washed in 500 μl phosphate-buffered saline (PBS), shaken at 1,500 rpm for 2–5 min, and centrifuged at 15,000 rpm for 5 min. Supernatant was removed and pellets were resuspended in 200 μl PBS. DNA quality was assessed by 1% agarose gel electrophoresis in 0.5 × TAE buffer stained with GelRed (10 μl/100 mL). Samples (10 μl DNA + 2 μl Orange Loading Dye) were run with a 1.5 kb DNA ladder at 100 V for 40 min. DNA purity was measured by NanoDrop (260/280), and concentration by Qubit 2.0 fluorometer.

Quantitative PCR (qPCR) analyses were performed on endpoint samples across 15 °C–40 °C. The protocol was adapted from a previously described RT-qPCR method for fungal quantification (Lanubile et al., 2017), with modifications for gDNA and absolute quantification. Species-specific primers targeting the calmodulin gene were designed from reference sequences retrieved from GenBank (Sayers et al., 2025) (NCBI accession numbers AY974341.1, MT010888, PP831809) using Primer3 software (Untergasser et al., 2012). Primer sequences are listed in Table 1. Specificity was verified by conventional PCR using gDNA from target and non-target species; amplicons matched expected sizes and no cross-amplification was detected. Each biological replicate (*n* = 3) was analysed in technical duplicate. Reactions contained 20 ng template DNA in 20 μl using FluoCycle™II SYBR Green Master Mix (EuroClone) on a Bio-Rad CFX96 system. Cycling conditions were 95 °C for 3 min; 35 cycles of 95 °C for 10 s and 58 °C for 20 s; followed by melt-curve analysis from 60 °C to 95 °C in 0.5 °C increments. No-template controls were included for each primer pair and showed no amplification. Melt curves from positive samples indicated a single specific product. Absolute quantification used standard curves from fivefold serial dilutions of genomic DNA for each target species (starting at 20 ng DNA). Cq values were converted to calmodulin gene copy number (GCN) using the corresponding standard curve.

**Table 1:** Species-specific calmodulin primer pairs used for qPCR quantification in mixed cultures.

Species	Primer	Sequence (5^′^–3^′^)	bp
*A. flavus*	AfCaM F	GGCCGACTCTTTGACTGAAG	166
*A. flavus*	AfCaM R	CCATCACCGTCCTTGTCCTA	166
*F. verticillioides*	FvCaM F	CCATAGGCCGACTCACTTACT	155
*F. verticillioides*	FvCaM R	CCATCGCCATCCTTGTCCTA	155
*F. graminearum*	FgCaM F	CAGGACATGATCAACGAGGTC	150
*F. graminearum*	FgCaM R	TTAGCGTTGCCACTCTACGG	150

Endpoint DNA-based species contributions in mixed cultures were calculated from absolute calmodulin GCN estimates obtained by standard-curve quantification, expressed as each species' proportion of the summed GCN within that sample (Reyes-Calderón et al., 2020):


$$\begin{array}{l} p_{i}=\frac{C_{i}}{\sum_{j}C_{j}}, \end{array}$$

where *C*_*i*_ is the estimated GCN for species *i* and the sum extends over all species present in that culture. For each sample, *p*_*i*_ was expressed as a percentage of total fungal DNA (sum = 100% per sample) and summarised as mean ± SD across biological replicates (*n* = 3).

### 2.7 Statistical analysis

Analyses were performed in Python 3.11 using SciPy, pandas, NumPy, and statsmodels (for *post-hoc* multiple comparisons); figures were generated using matplotlib and seaborn. Optical density data are reported as mean ± SD across wells (*n* = 12 per treatment–temperature combination). In mixed cultures, OD-derived metrics (including logistic parameters, ΔOD_max_, and ΔNPI) were interpreted as culture-level optical descriptors, whereas species-level inference was based primarily on endpoint qPCR-derived GCN and DNA-based proportional contribution.

For logistic fit quality, both the classical coefficient of determination (*R*^2^) and a study-specific empirical score (*R*^2*^) were reported. Classical *R*^2^ was calculated as:


$$\begin{array}{l} R^{2}=1-\frac{SS_{\text{res}}}{SS_{\text{tot,mean}}},\;SS_{\text{tot,mean}}=\overset{n}{\underset{i=1}{\sum }}{\left(y_{i}-ȳ\right)}^{2}, \end{array}$$

where *y*_*i*_ are observed ΔOD values and ȳ is their mean. Because *R*^2^ can be misleading in nonlinear regression and should not be used as a sole criterion (Spiess and Neumeyer, 2010; Kvålseth, 1985), an empirical score aligned with baseline-corrected trajectories was additionally computed:


$$\begin{array}{l} R^{2*}=\max\left(0,1-\frac{SS_{\text{res}}}{SS_{\text{tot,baseline}}}\right)\times \min\left(1,{\left(\frac{\text{Δ}OD_{\text{range}}}{0.25}\right)}^{2}\right), \end{array}$$

with


$$\begin{array}{l} SS_{\text{tot,baseline}}=\overset{n}{\underset{i=1}{\sum }}{\left(y_{i}-y_{0}\right)}^{2},\text{ Δ}OD_{\text{range}}=\max\left(y_{i}\right)-\min\left(y_{i}\right), \end{array}$$

where *y*_0_ is the first time point. To separate biologically negligible trajectories from informative growth curves, a predefined signal criterion was applied: Δ*OD*_max_ ≥ 0.10. Fits with Δ*OD*_max_ < 0.10 were classified as low-signal and logistic parameters were not interpreted. For signal-positive curves, NRMSE (RMSE normalised by Δ*OD*_max_) was also reported as a scale-independent error metric, consistent with recommendations to combine multiple diagnostics and biological plausibility in nonlinear growth modelling (Archontoulis and Miguez, 2015; Sprouffske and Wagner, 2016). Logistic parameters were interpreted only when both criteria were satisfied: Δ*OD*_max_ ≥ 0.10 and *R*^2*^ ≥ 0.95. Fit quality metrics for all treatment–temperature combinations are reported in Supplementary Figure 3.

At each incubation temperature, differences among treatments in endpoint ΔOD_max_ were tested using one-way ANOVA on per-well values (*n* = 12 wells per treatment). When significant, pairwise comparisons were performed using Tukey's HSD test (α = 0.05). Compact letter display and overall ANOVA *p*-values are reported in Supplementary Figure 4.

For qPCR data, log_2_ fold change (co-culture vs. monoculture) was calculated for each species, combination, and temperature. Group differences were tested using Welch's two-sample *t*-test (unequal variances; *n* = 3 biological replicates per group). Exact *p*-values and significance classes (ns, *, **, and ***) are reported in Supplementary Figure 7.

## 3 Results

### 3.1 Growth curve and logistic growth model fitting

**Figure 1:**
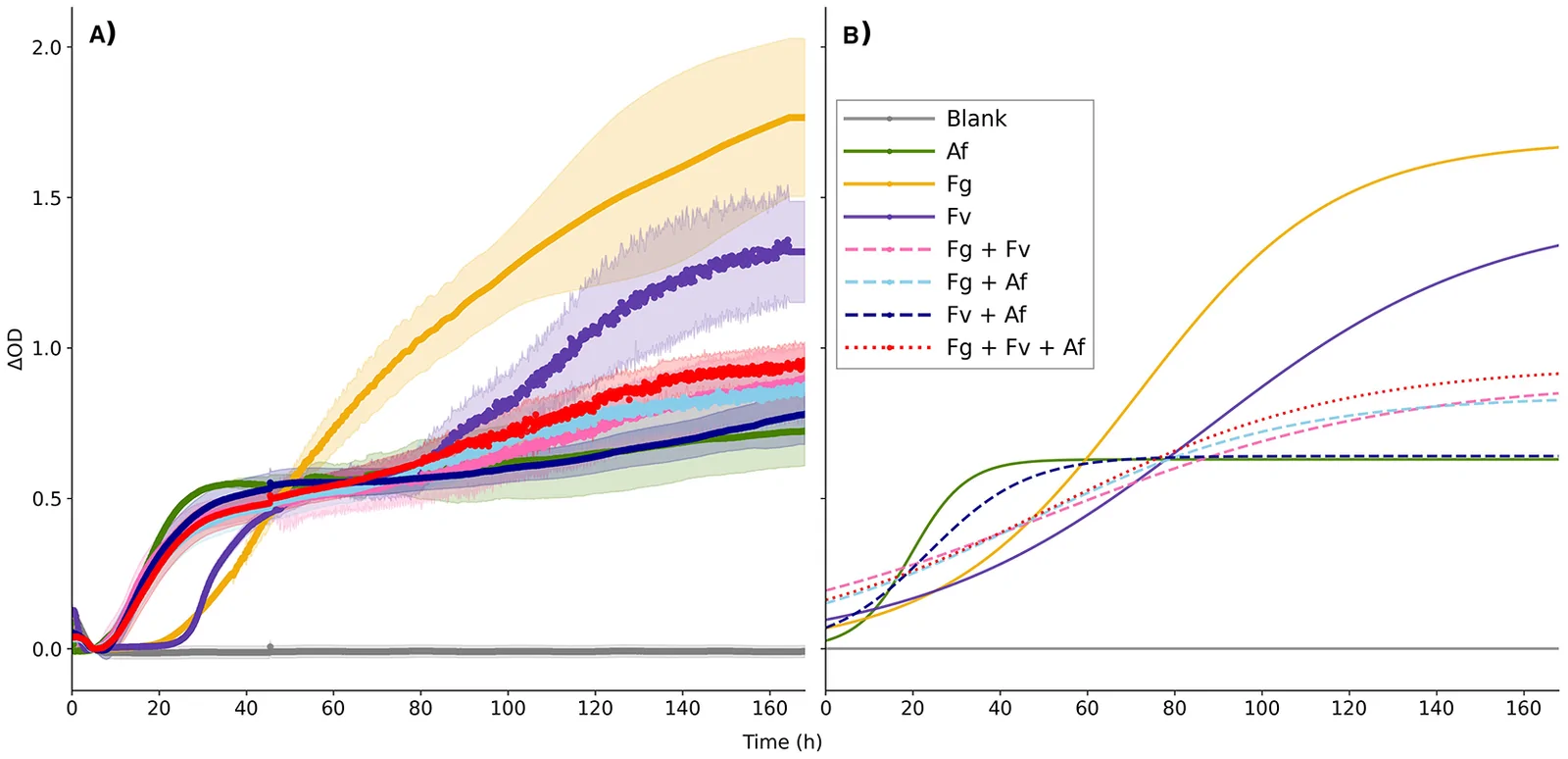
Growth curves and logistic model fitting at 20 °C. **(A)** Baseline-corrected optical density (ΔOD) over time, shown as mean ± SD (*n* = 12). **(B)** Fitted logistic growth curves for the same treatments. Solid lines represent monocultures, dashed lines dual cultures, and the dotted line the three-species culture. Line colours distinguish species and culture combinations as indicated in the legend.

**Figure 2:**
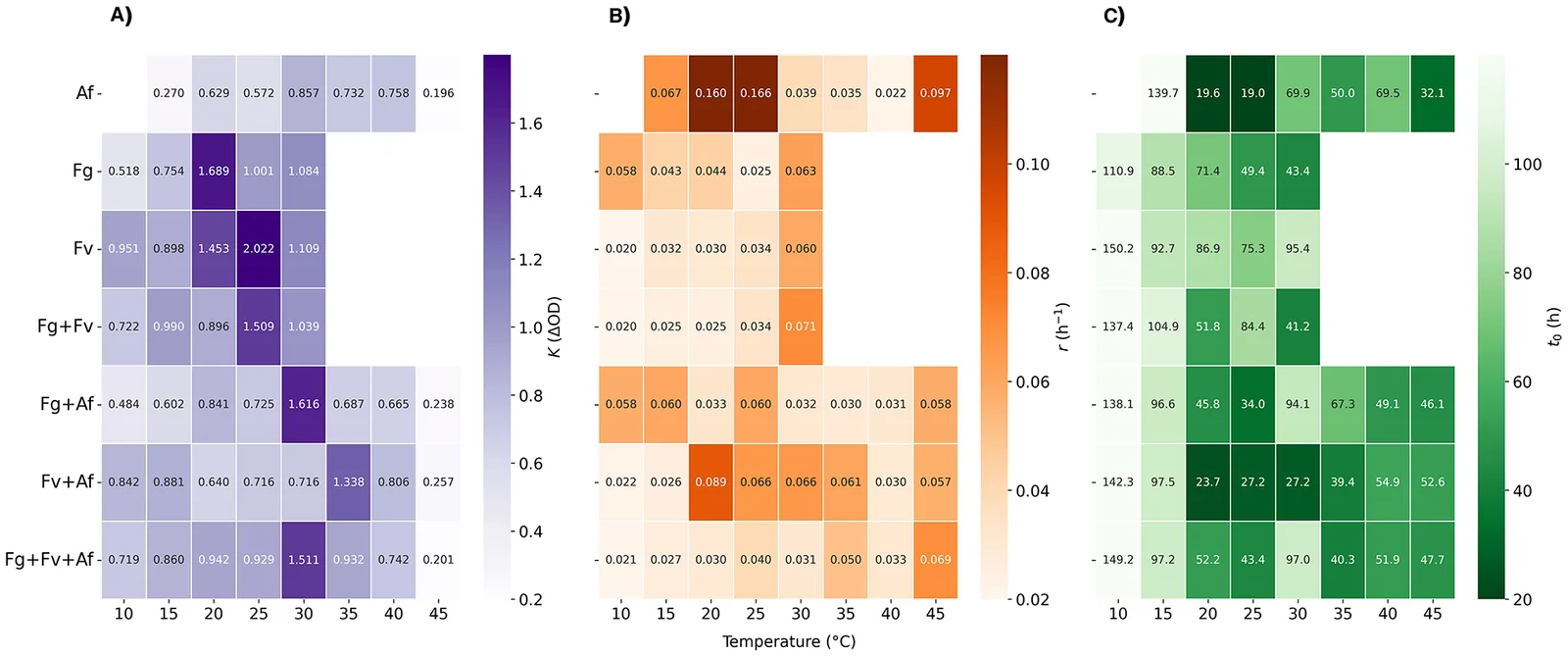
Parameters derived from three-parameter logistic fits to baseline-corrected optical density time series. Heatmaps show **(A)** carrying capacity (*K*), **(B)** growth rate (*r*), and **(C)** inflection point (*t*_0_) for monocultures, dual cultures, and the three-species culture. Cells are coloured by parameter magnitude, with values annotated. Blank cells indicate conditions where parameters were not retained because growth was negligible and/or fit quality was below the predefined threshold (*R*^2*^ < 0.95; see Supplementary material).

Baseline-corrected optical density time series followed sigmoidal growth trajectories across monocultures, dual cultures, and the three-species culture. As exemplified at 20 °C (Figure 1A), species differed in both growth magnitude and timing, for example *F. graminearum* and *F. verticillioides* reached higher maximum ΔOD values than *A. flavus*, while dual and triple cultures fell between or below their constituent monocultures. Logistic model fits captured the observed dynamics closely (Figure 1B), with the majority of fits meeting the predefined fit-quality criterion (*R*^2*^ ≥ 0.95). Fitted parameters, carrying capacity (*K*), growth rate (*r*), and inflexion point (*t*_0_), were extracted for each treatment across 10 °C–45 °C and are summarised in Figure 2. In mixed cultures, these OD-derived parameters are interpreted as empirical descriptors of whole-culture optical dynamics. Validation of all 56 treatment–temperature combinations is reported in Supplementary Figure 3. Of these, 46 models met the acceptance criteria and were retained for interpretation of *K*, *r*, and *t*_0_. Logistic parameters were not reported for treatments when fit quality criteria were not met (growth was negligible and/or *R*^2*^ ≤ 0.95, see Supplementary material).

### 3.2 Temperature dependence of logistic growth parameters

In monoculture, *F. verticillioides* reached its highest carrying capacity at 25 °C (*K* = 2.02) and *F. graminearum* at 20 °C (*K* = 1.69). *A. flavus* peaked at 30 °C (*K* = 0.86) with a broader thermal range, retaining *K* = 0.76 at 40 °C (Figure 2A). In dual cultures, *F. graminearum* + *F. verticillioides* peaked at 25 °C (*K* = 1.51) but declined sharply above 30 °C, consistent with the reduced monoculture optical performance of both species at higher temperatures. *F. graminearum* + *A. flavus* reached a higher *K* at 30 °C (*K* = 1.62), and *F. verticillioides* + *A. flavus* peaked at 35 °C (*K* = 1.34). The three-species culture peaked at 30 °C (*K* = 1.51) and maintained *K* ≥ 0.74 up to 40 °C.

For growth rate, *A. flavus* in monoculture exhibited the highest values, peaking at 20 °C–25 °C (*r* = 0.16), while *F. graminearum* and *F. verticillioides* both peaked at 30 °C (*r* = 0.06) (Figure 2B). In dual cultures, *F. graminearum* + *F. verticillioides* had consistently low *r* values across all temperatures (*r* ≤ 0.03), whereas *F. graminearum* + *A. flavus* maintained moderate rates between 15 °C and 25 °C (*r* = 0.03–0.06). *F. verticillioides* + *A. flavus* reached the highest dual-culture *r* at 20 °C (*r* = 0.09) and retained *r* = 0.06–0.07 between 25 °C and 35 °C. The three-species culture showed intermediate rates, with *r* = 0.07 at 45 °C and *r* = 0.05 at 35 °C.

The inflexion point *t*_0_ was lowest at intermediate temperatures and increased towards thermal extremes (Figure 2C). In monoculture, *A. flavus* reached minimum *t*_0_ at 20 °C–25 °C (*t*_0_ = 19.05–19.61 h), whereas *F. graminearum* (*t*_0_ = 49.39–71.40 h) and *F. verticillioides* (*t*_0_ = 75.28–86.87 h) required substantially longer at the same temperatures. At 10 °C–15 °C, all monocultures showed delayed inflexion points (*t*_0_>88 h), consistent with reduced metabolic activity. In dual cultures, *F. graminearum* + *A. flavus* had lower *t*_0_ than *F. graminearum* alone at 25 °C (*t*_0_ = 34.03 vs. 49.39 h), and *F. verticillioides* + *A. flavus* showed reduction relative to *F. verticillioides* alone at 20 °C (*t*_0_ = 23.65 vs. 86.87 h). In contrast, *F. graminearum* + *A. flavus* at 30 °C exhibited a higher *t*_0_ (94.14 h) than either monoculture at the same temperature (*F. graminearum*: 43.43 h; *A. flavus*: 69.87 h). *F. graminearum* + *F. verticillioides* followed its component species closely, with *t*_0_ ranging from 41.25 h at 30 °C to 137.39 h at 10 °C. The three-species culture showed intermediate values at most temperatures (e.g., *t*_0_ = 43.38 h at 25 °C, 97.01 h at 30 °C).

### 3.3 Maximum growth across the temperature gradient

**Figure 3:**
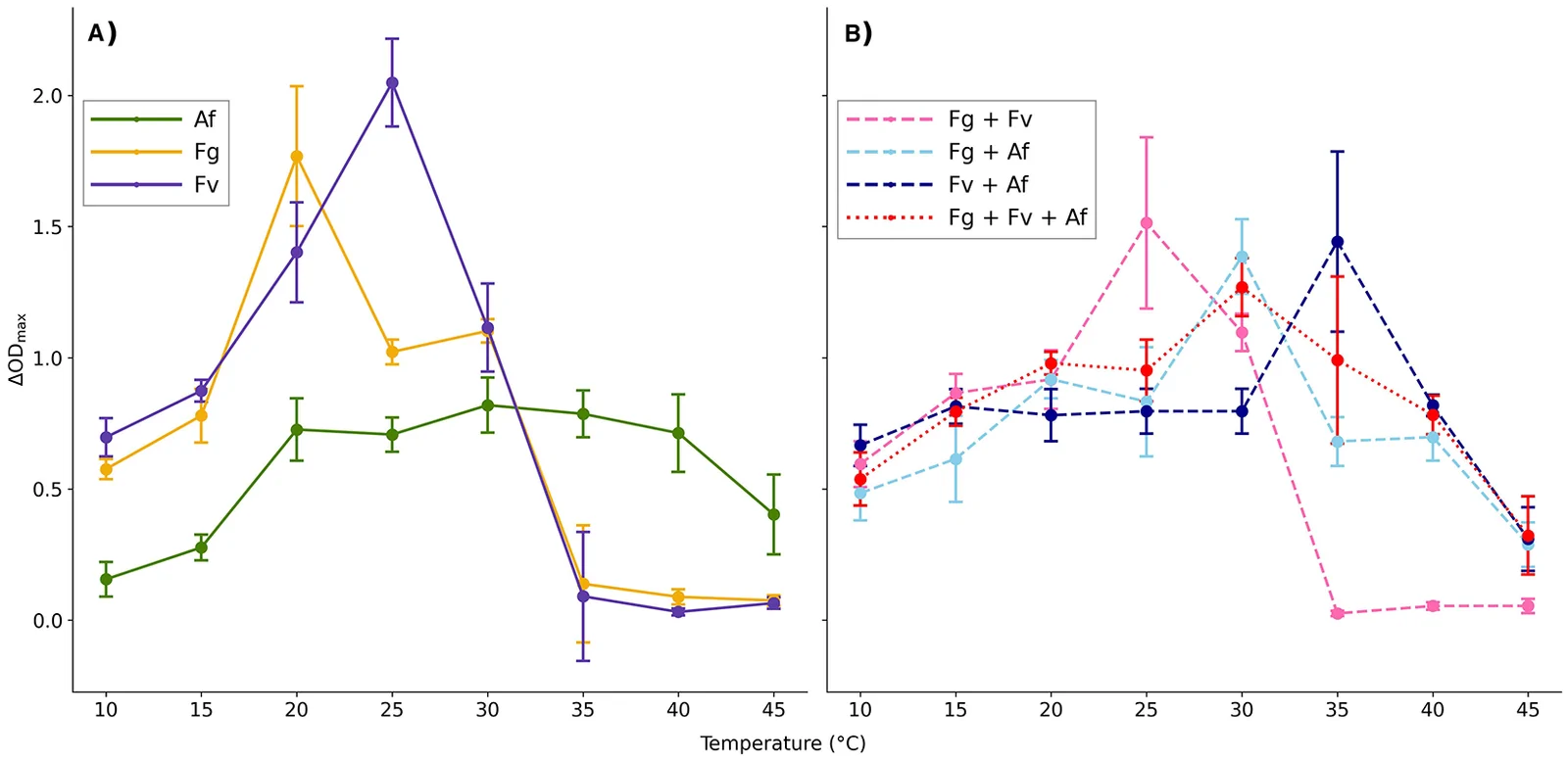
Maximum growth (ΔOD_max_) of **(A)** monocultures and **(B)** dual- and three-species cultures across the temperature gradient (10 °C–45 °C). In mixed cultures, ΔOD_max_ reflects the total optical signal of the culture. Points represent mean ± SD (*n* = 12), with connecting lines shown as visual guides. Solid lines indicate monocultures, dashed lines dual cultures, and the dotted line the three-species culture.

Maximum baseline-corrected optical density (ΔOD_max_) was recorded for each treatment after 168 h of incubation across the temperature range. In monoculture (Figure 3A), *F. verticillioides* reached the highest ΔOD_max_ at 25 °C (2.04), followed by *F. graminearum* at 20 °C (1.77); both species declined sharply at ≥ 35 °C (ΔOD_max_ ≤ 0.09). *A. flavus* peaked at 30 °C (0.82) and maintained moderate growth up to 40 °C (0.70), with reduced but detectable growth at 45 °C (0.27). In dual cultures, peak ΔOD_max_ shifted depending on the species combination (Figure 3B). *F. graminearum* + *F. verticillioides* peaked at 25 °C (1.51) and declined above 30 °C, consistent with the reduced monoculture optical signal of both species at higher temperatures. *F. graminearum* + *A. flavus* peaked at 30 °C (1.38), while *F. verticillioides* + *A. flavus* peaked at 35 °C (1.44), exceeding all monoculture values at that temperature. At ≥ 35 °C, only combinations containing *A. flavus* sustained substantial growth. The three-species culture grew across the full 10 °C–45 °C range and reached its highest ΔOD_max_ at 30 °C (1.27).

### 3.4 Interaction effects on growth performance in co-cultures

**Figure 4:**
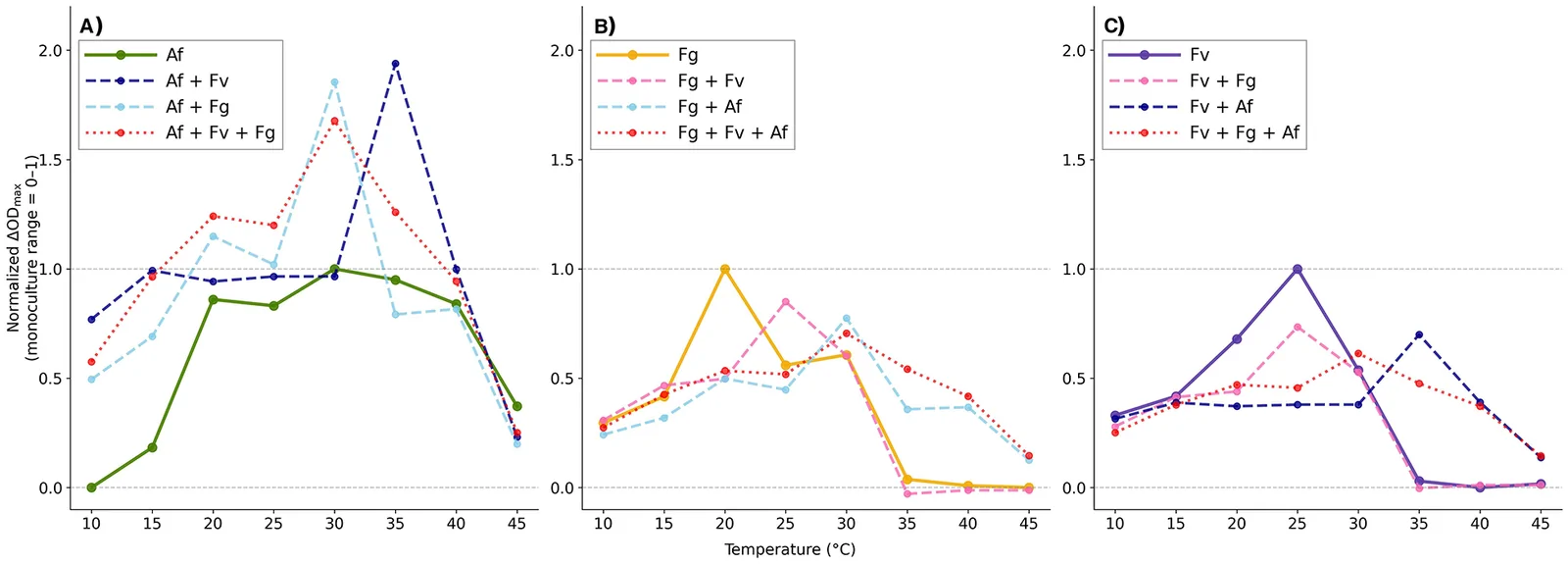
Normalised maximum optical performance of cultures scaled to the monoculture reference range of **(A)** *A. flavus*, **(B)** *F. graminearum*, and **(C)** *F. verticillioides* in monoculture, dual, and three-species cultures across the temperature gradient (10 °C–45 °C). For each focal species, ΔOD_max_ values were normalised to the monoculture range across all temperatures (minimum = 0, maximum = 1). Co-culture values were scaled using the same species-specific reference. Values above 1 indicate optical signal above the monoculture maximum, and values below 0 indicate optical signal below the monoculture minimum. Dashed horizontal lines mark monoculture bounds (0–1). Points represent means across replicates (*n* = 12), and connecting lines are shown as visual guides. This index is a comparative optical performance metric and not a direct species-specific growth estimate in mixed culture.

Co-culture effects were quantified as ΔNPI, the difference between normalised maximum growth in co-culture and monoculture at the same temperature. Positive values indicate higher co-culture optical performance, and negative values indicate lower co-culture optical performance, relative to monoculture at the same temperature (Figure 4, summary table provided in Supplementary material). Since *F. graminearum* and *F. verticillioides* showed minimal monoculture growth at ≥ 35 °C (Figure 3), co-culture outcomes at these temperatures should be interpreted cautiously as they might largely reflect the contribution of *A. flavus*.

The total optical signal of co-cultures containing *A. flavus* exceeded the corresponding monoculture reference range in multiple cases (Figure 4A). The strongest effect was observed with *F. verticillioides* at 35 °C (ΔNPI = 0.99), followed by *F. graminearum* at 30 °C (ΔNPI = 0.85) and *F. verticillioides* at 15 °C (ΔNPI = 0.81). The triple culture yielded positive ΔNPI at all temperatures except 45 °C, peaking at 15 °C (ΔNPI = 0.78). Suppressions were minor, mainly at 45 °C across all co-cultures (ΔNPI = −0.12 to −0.17) and at 35 °C with *F. graminearum* (ΔNPI = −0.16).

In cultures containing *F. graminearum*, both increases and decreases in normalised optical performance relative to the monoculture reference were observed (Figure 4B). Co-culture with *F. verticillioides* produced the largest enhancement at 25 °C (ΔNPI = 0.29), with negligible effects at other temperatures. Co-culture with *A. flavus* resulted in suppression at 10 °C–25 °C (ΔNPI = −0.05 to −0.50) but shifted to enhancement at ≥ 30 °C (ΔNPI = 0.13–0.36). The three-species culture followed a similar pattern, with suppression at 20 °C–25 °C (ΔNPI = −0.47 and −0.04) and enhancement at ≥ 30 °C (ΔNPI = 0.10–0.50). The strongest suppressions across all co-cultures occurred at 20 °C (ΔNPI = −0.47 to −0.50).

When scaled to the *F. verticillioides* monoculture reference, co-cultures showed the broadest reduction in normalised optical performance (Figure 4C). The strongest negative effect occurred with *A. flavus* at 25 °C (ΔNPI = −0.62), with suppression also in the three-species culture at the same temperature (ΔNPI = −0.54). Co-culture with *F. graminearum* had comparatively modest effects across the temperature range (ΔNPI: −0.27 to 0.01). At ≥ 35 °C, all co-cultures shifted to enhancement, with the largest values in dual culture with *A. flavus* at 35 °C (ΔNPI = 0.67) and in the three-species culture at the same temperature (ΔNPI = 0.45).

### 3.5 Quantification of individual species contribution in mixed cultures

**Figure 5:**
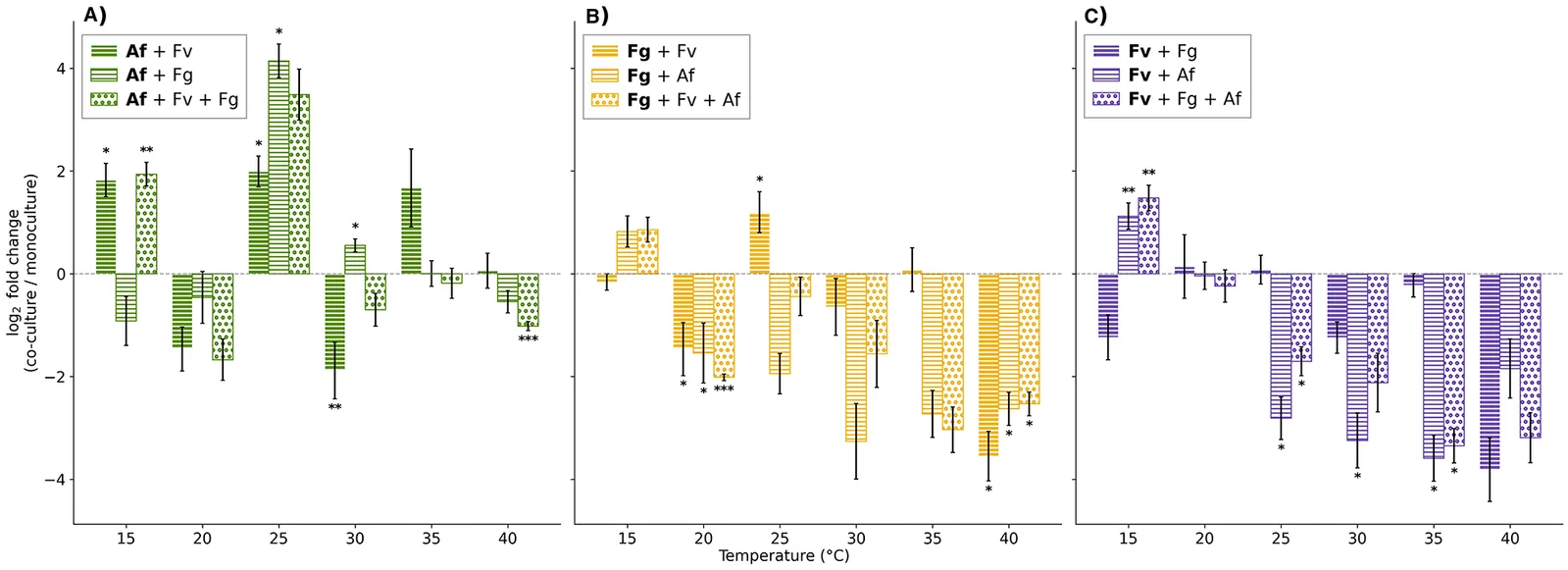
log_2_ fold change of calmodulin gene copy number (GCN) in co-culture relative to monoculture for **(A)** *A. flavus*, **(B)** *F. graminearum*, and **(C)** *F. verticillioides* across 15 °C–40 °C at 168 h. Bars represent mean log_2_ fold change (co-culture/monoculture). Error bars show propagated uncertainty from both co-culture and monoculture biological replicates (*n* = 3 per group). The dashed line at *y* = 0 indicates no change relative to monoculture. Significance was assessed by Welch's two-sample *t*-test: ^*^p < 0.05, ^**^p < 0.01, and ^***^p < 0.001. Exact *p*-values are reported in Supplementary Figure 7.

Co-culture effects on species-specific gene copy number (GCN) were assessed as endpoint log_2_ fold change relative to monoculture (Figure 5; absolute GCN values and full statistical results are provided in Supplementary material). At 15 °C, *A. flavus* increased significantly when paired with *F. verticillioides* (log_2_FC = +1.82, *p* < 0.05) and in the three-species combination (log_2_FC = +1.94, *p* < 0.01). The largest increase was observed at 25 °C, where *A. flavus* increased with both *F. graminearum* (log_2_FC = +4.14, *p* < 0.05) and *F. verticillioides* (log_2_FC = +1.99, *p* < 0.05). At 30 °C, *A. flavus* remained higher with *F. graminearum* (log_2_FC = +0.55, *p* < 0.05), but decreased with *F. verticillioides* (log_2_FC = −1.88, *p* < 0.01). At 40 °C, a significant decrease was detected only in the three-species combination (log_2_FC = −1.02, *p* < 0.001).

*F. graminearum* generally showed reduced GCN in co-culture. Significant decreases were detected at 20 °C with *A. flavus* (log_2_FC = −1.54, *p* < 0.05), with *F. verticillioides* (log_2_FC = −1.47, *p* < 0.05), and in the three-species combination (log_2_FC = −2.02, *p* < 0.001). At 25 °C, *F. graminearum* increased with *F. verticillioides* (log_2_FC = +1.20, *p* < 0.05), while values were lower but not significant with *A. flavus* (log_2_FC = −1.94, *p* = 0.10). At ≥ 35 °C, all co-cultures showed reduced *F. graminearum* GCN.

*F. verticillioides* increased at 15 °C in dual culture with *A. flavus* (log_2_FC = +1.12, *p* < 0.01) and in the three-species combination (log_2_FC = +1.47, *p* < 0.01). From 25 °C onward, *F. verticillioides* was progressively reduced, including significant decreases with *A. flavus* at 25 °C, 30 °C, and 35 °C (log_2_FC = −2.81, −3.25, and −3.59; all *p* < 0.05). In the three-species combination, significant decreases were also observed at 25 °C and 35 °C (log_2_FC = −1.70 and −3.35; *p* < 0.05). Co-culture with *F. graminearum* alone did not yield significant changes across temperatures, despite negative fold-change trends above 25 °C.

**Figure 6:**
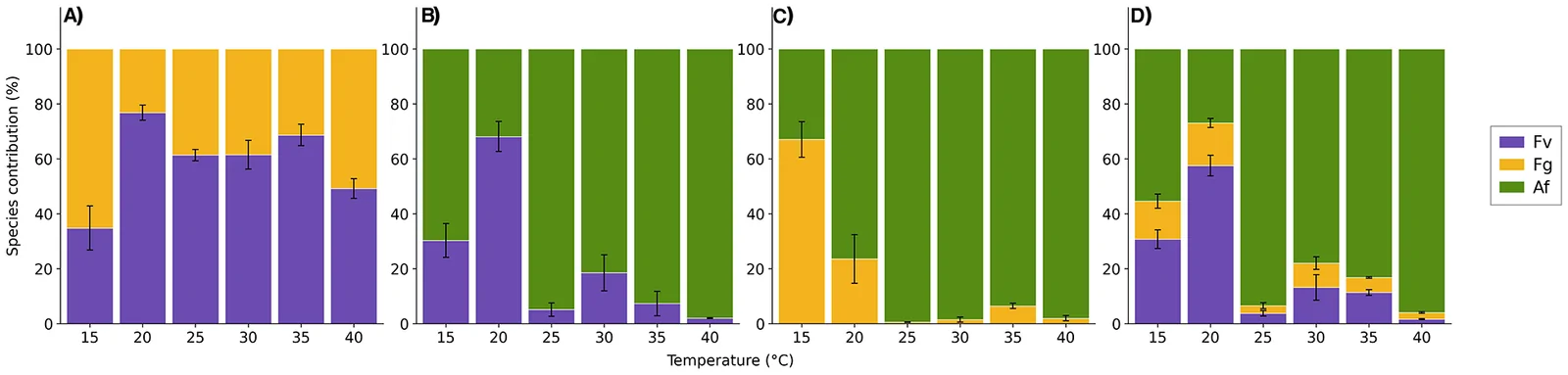
Proportional species contributions to total fungal DNA in **(A)** *F. graminearum + F. verticillioides*, **(B)** *A. flavus + F. verticillioides*, **(C)** *A. flavus + F. graminearum*, and **(D)** the three-species culture across 15 °C–40 °C at the end of the incubation period (168 h). Contributions were calculated from species-specific calmodulin gene copy number (GCN) as percentages of total fungal DNA per sample (sum = 100% per sample), then summarised as mean ± SD across biological replicates (*n* = 3). These proportions are endpoint DNA-based estimates and do not necessarily represent biomass or metabolic activity.

To summarise endpoint community composition, species contributions were expressed as percentages of total fungal DNA per sample (Figure 6). In *F. graminearum + F. verticillioides*, dominance shifted from *F. graminearum* at 15 °C ( 65%) to *F. verticillioides* at 20 °C ( 77%); from 25 °C to 35 °C, *F. verticillioides* remained higher (58%–69%), and at 40 °C both species were near-equal ( 51% vs. 49%). In *F. verticillioides + A. flavus, A. flavus* was dominant at 15 °C ( 70%) and from 25 °C onward (> 81%), reaching 98% at 40 °C; only at 20 °C did *F. verticillioides* prevail ( 68%). In *F. graminearum + A. flavus, F. graminearum* was higher at 15 °C ( 67%), but *A. flavus* dominated from 20 °C onward (76%–99%). In the three-species combination, *A. flavus* was the major contributor at almost all temperatures ( 55% at 15 °C to 96% at 40 °C), while *F. verticillioides* peaked at 20 °C ( 57%). *F. graminearum* accounted for the smallest fraction, ranging from 15% at 20 °C to 2% at 40 °C.

## 4 Discussion

Microplate spectrophotometric measurements have been used for decades as an indirect approach to quantify filamentous fungal growth in liquid cultures, based on the relationship between optical signal and mycelial accumulation (Granade et al., 1985; Langvad, 1999; Slowik et al., 2023). Still, most applications focus on single-species assays and endpoint measurements, providing limited information on growth dynamics or species interactions. In the present study, this approach was extended by combining continuous optical monitoring with logistic growth modelling and species-specific qPCR to quantify growth kinetics and endpoint species contributions within mixed cultures. The integration of these three approaches provided complementary information that no single method could provide alone, helping to distinguish culture-level optical dynamics from endpoint species composition. Overall, this framework showed that mixed-culture outcomes were shaped by thermal physiology, growth strategy, and endpoint competitive dominance.

Temperature is a major abiotic determinant of fungal performance and distribution (Perrone et al., 2020), and defining the thermal growth range of each species is essential to understand niche overlap and potential competitive interactions. In monoculture, growth responses across the temperature gradient were consistent with established ecophysiological patterns (Magan et al., 2011). *A. flavus* maintained measurable growth throughout the full range (10 °C–45 °C) and showed relatively higher growth under warm and hot conditions, while *F. graminearum* and *F. verticillioides* showed narrower thermal optima and decreased above 35 °C. The growth of *A. flavus* at elevated temperatures has been associated with the activation of heat-shock and oxidative-stress pathways (Medina et al., 2014), while *Fusarium* spp. exhibit reduced tolerance to heat stress, including slower recovery of mycelial growth and lower production of protective metabolites, but higher tolerance to cold stress (Vujanovic et al., 2012; Blacutt et al., 2018).

Fitting logistic growth curves provided an empirical description of OD trajectories through *K*, *r*, and *t*_0_. Three-parameter logistic models have previously been used to summarise optical density-based growth trajectories in microbial and filamentous fungal systems (Sprouffske and Wagner, 2016; Simpson et al., 2022; Lo Grasso et al., 2023). In this context, these parameters should be interpreted as descriptors of culture-level optical dynamics, especially in mixed cultures. The estimated parameters did not vary in parallel between species or culture conditions, indicating that the processes controlling the final accumulation of biomass and early colonisation respond differently to temperature and co-culture. In monoculture, both *Fusarium* species generally reached higher *K* values, suggesting greater final culture density under favourable conditions, but exhibited lower *r* and later *t*_0_, indicating slower establishment. In contrast, *A. flavus* showed lower *K* but higher *r* and earlier *t*_0_, reflecting rapid early expansion despite lower final biomass (Prosser, 1995). Accordingly, *K*, *r*, and *t*_0_ capture distinct aspects of culture-level performance: carrying capacity describes final optical accumulation, growth rate describes early expansion, and the inflexion point (*t*_0_) describes the timing of transition into rapid growth. In our dataset, *t*_0_ was lowest under intermediate temperatures and increased at 10 °C and 45 °C (Figure 2C), whereas peak *K* and *r* varied with species and culture type (Figures 2A, B); these parameters are therefore most informative when interpreted jointly (Baranyi and Roberts, 1994; Fukami, 2015).

Across co-cultures, interaction outcomes were strongly temperature dependent, but the interpretation differed between OD-based culture-level performance and DNA-based qPCR endpoint species composition. This distinction is particularly important in mixed filamentous fungal cultures, where total OD cannot be directly assigned to individual species because optical signal is affected by mycelial morphology and light scattering (Meletiadis et al., 2001; Hameed et al., 2024). At lower temperatures, the combined OD and qPCR evidence indicated more balanced interaction outcomes, including conditions where *Fusarium* species remained competitive. At 15 °C, the total optical signal of co-cultures containing *A. flavus* exceeded the corresponding monoculture reference range, and endpoint qPCR showed increased *A. flavus* GCN in several mixed cultures. This suggests that, near the lower thermal range of *A. flavus*, the presence of other fungi may have favoured its final DNA-based contribution, although the underlying mechanism remains unknown. Similar context dependence has been reported in saprobic soil fungi, where the thermal responses of individual species were reshaped by the presence of co-occurring fungi, indicating that species interactions can modulate how a species performs near its thermal limits (Lammel et al., 2023).

At 20 °C–25 °C, the divergence between OD and qPCR results became particularly informative. Although some co-cultures showed elevated total optical signal, endpoint qPCR showed that this signal was not equally attributable to all species (Figures 5, 6), indicating that culture-level OD reflects combined mycelial scattering rather than species-specific abundance (Meletiadis et al., 2001; Slowik et al., 2023; Hameed et al., 2024). In the *F. graminearum + F. verticillioides* pairing, increased optical performance at 25 °C was supported by increased *F. graminearum* GCN, indicating a positive endpoint response in that combination. By contrast, in pairings involving *A. flavus*, qPCR showed a strong increase in the final DNA-based contribution of *A. flavus*, while both *Fusarium* species were reduced relative to their monocultures. This highlights a central limitation of OD measurements in mixed cultures, as they describe the behaviour of the culture as a whole, but do not identify which species contributes to the signal.

At warmer temperatures, especially 30 °C–40 °C, qPCR showed a progressive increase in the endpoint DNA-based contribution of *A. flavus* in most mixed cultures, accompanied by reduced contributions from both *Fusarium* species. Unlike at 20 °C–25 °C, co-culture OD and endpoint qPCR results were more consistent under these conditions, with strong optical performance in *A. flavus*-containing co-cultures reflecting endpoint dominance by *A. flavus*. This was particularly evident at 35 °C–40 °C, where *Fusarium* species were close to or beyond their favourable thermal range, whereas *A. flavus* maintained measurable growth (Giorni et al., 2009), (Satterlee et al., 2024).

Taken together, these results indicate asymmetric, temperature-dependent interaction outcomes. Cooler conditions allowed more balanced or *Fusarium*-competitive states, whereas warmer conditions favoured increasing *A. flavus* dominance in endpoint DNA-based composition. Under the tested conditions, this pattern is best described as apparent competitive displacement at higher temperature. Such non-reciprocal responses are common in multispecies fungal systems, where competitive outcomes depend on growth strategy, resource acquisition and environmental stress (Prosser, 1995; Fukami, 2015). The progressive increase in *A. flavus* at warmer temperatures is best interpreted in relation to the monoculture thermal responses and the rapid colonisation strategy reflected by higher *r* and earlier *t*_0_ described above. This may have provided an advantage at warmer temperatures where both *Fusarium* species were physiologically constrained. Together with field evidence that co-infection by *A. flavus* and *Fusarium* spp. can modify fungal incidence and toxin profiles (Giorni et al., 2019), these findings suggest that warming may reorganise toxigenic fungal communities by shifting competitive balance instead of replacing one species with another.

The present framework offers several directions for further development. The strains selected for this study were cereal-associated isolates from the ISPA-CNR collection previously used in ecophysiological work. Comparisons among multiple *A. flavus* and *F. verticillioides* strains have shown that inter-species differences in growth-related responses are generally greater than inter-strain variation, whereas strain effects are more evident for mycotoxin production than for growth (Giorni et al., 2009; Jurado et al., 2008; Lazzaro et al., 2012). Since this study focused on temperature-dependent growth kinetics and endpoint species composition rather than toxin biosynthesis, these isolates provide a defined starting point for comparing mixed-culture behaviour among the three target species. We nevertheless acknowledge that future studies with multiple isolates per species should add robustness to the observed interaction patterns. The workflow could also be extended to crop-derived substrates or host tissues (Giorni et al., 2019), and to combined temperature and water-activity gradients (Medina et al., 2015; Mannaa and Kim, 2017; Ribeiro et al., 2006). Direct mycotoxin quantification was not included, as the experiment was designed for growth monitoring and endpoint species composition in small culture volumes. Future work could address this through scaled-up experiments (Calvo et al., 2002; Gallo et al., 2016; Huang et al., 2023) or gene-expression assays targeting key biosynthetic regulators (e.g., *aflR, aflD, FUM1*, and *TRI5*) within the microplate format (Yu et al., 2004; Wang et al., 2022; Lanubile et al., 2021). Refinements of the molecular component could further improve resolution of metabolically active biomass through RNA-based approaches (Anderson and Parkin, 2007; Tellenbach et al., 2010; Wallander et al., 2013).

In summary, integrating high-frequency microplate monitoring with logistic growth curve fitting and species-specific qPCR enabled quantitative comparison of temperature-dependent mixed-culture behaviour and endpoint species composition in three major cereal-associated fungi. The data indicate that *A. flavus* can strongly shape endpoint community composition in mixed cultures, with its contribution increasing with temperature and progressively reducing the endpoint DNA-based contribution of both *Fusarium* species above 25 °C and achieving near-complete dominance at 40 °C. These results are relevant for mycotoxin risk contexts in which *Aspergillus* and *Fusarium* species increasingly co-occur in cereal systems (Chen et al., 2023; Penagos-Tabares et al., 2025), and where warming is expected to favour thermotolerant *Aspergillus* populations and expand overlap with *Fusarium* spp. in temperate regions (Battilani et al., 2016; Casu et al., 2024). The resulting dataset provides quantitative descriptors that could be used to connect physiological responses to community-level outcomes in process-based models. Overall, the workflow presented here is transferable to other pathosystems and can be adapted to other fungal species or species combinations by substituting species-specific primer sets, integrating expression assays, or incorporating additional environmentally relevant variables.

## Data Availability

The original contributions presented in the study are included in the article/Supplementary material, further inquiries can be directed to the corresponding author/s.
