# Supplementary material for: How temperature reshapes interactions in mixed cultures of mycotoxigenic fungi
Source: Front Microbiol. 2026 Aug 24;17:1903639. doi: 10.3389/fmicb.2026.1903639 (PMC13547475; doi:10.3389/fmicb.2026.1903639)
Supplement: Supplementary file 1 [file Data_Sheet_1.pdf]

Supplementary Material

Supporting information for article "*How temperature reshapes interactions in mixed cultures of mycotoxigenic fungi*"

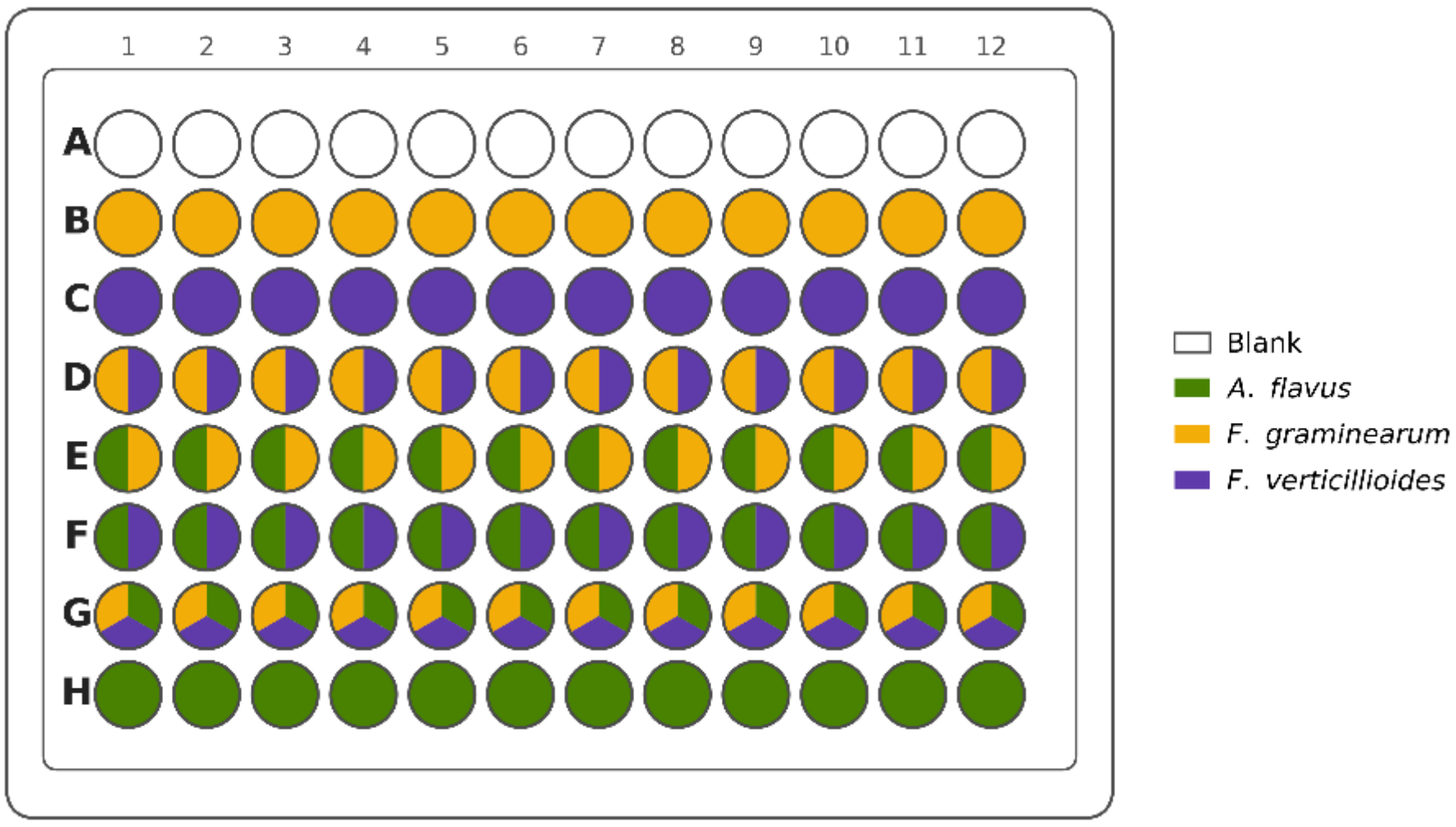

Supplementary Figure 1. Schematic layout of the 96-well microplate used for the co-culture assay. Rows were assigned to monocultures, dual-species combinations, the three-species culture, and a negative control (Blank). White wells indicate the negative control (100 µL medium + 100 µL sterile distilled water). For co-inoculations, fungal suspensions were mixed in equal proportions (50:50 for dual cultures; 33:33:33 for the three-species culture) to maintain the same total inoculum volume and spore concentration across treatments.

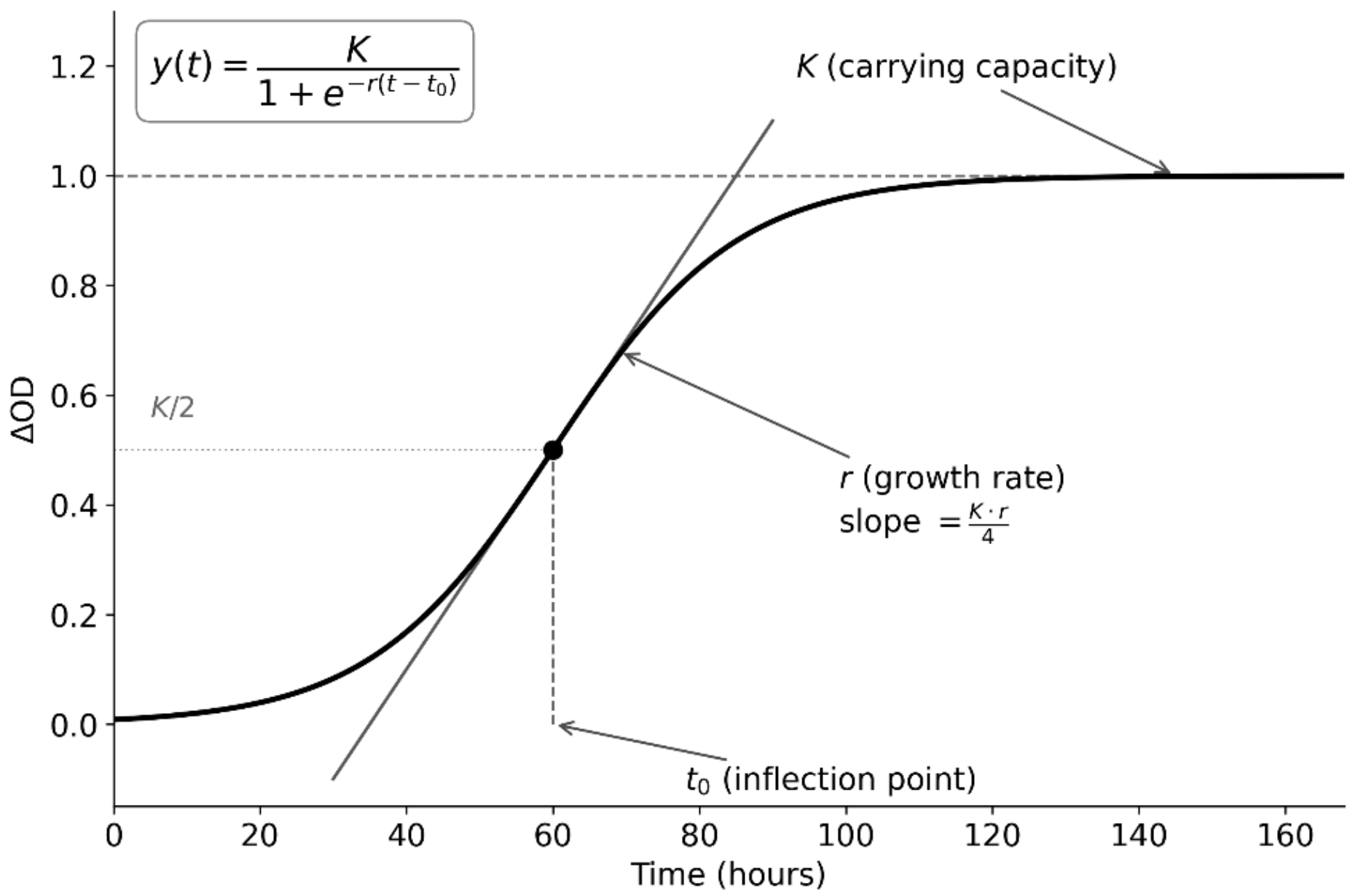

Supplementary Figure 2. Schematic representation of the three-parameter logistic growth model $y(t) = K / (1 + e\hat{\ }{-r(t - t_0)})$ fitted to baseline-corrected ΔOD time series by nonlinear least-squares regression. *K*: carrying capacity (maximum ΔOD); *r*: growth rate (steepness at inflection); t_0: inflection point (time at *K*/2, reflecting colonisation speed). The tangent line at t_0 illustrates the maximum slope ($K \cdot r / 4$).

		10 °C	15 °C	20 °C	25 °C	30 °C	35 °C	40 °C	45 °C
A. flavus	**R^2**	0.94	0.90	0.90	0.88	0.99	0.97	0.95	0.97
	**R^{2*}**	0.36†	0.97	0.99	0.99	1.00	1.00	0.99	1.00
	**NRMSE**	0.07	0.09	0.07	0.07	0.03	0.04	0.05	0.04
F. graminearum	**R^2**	0.98	0.98	0.99	0.91	0.99	0.97	0.20	0.05
	**R^{2*}**	0.99	1.00	1.00	0.99	1.00	0.14†	0.00†	0.00†
	**NRMSE**	0.05	0.04	0.04	0.07	0.03	0.05	0.12	0.08
F. verticillioides	**R^2**	0.91	0.98	0.97	0.99	0.99	0.38	0.32	0.30
	**R^{2*}**	0.97	0.99	0.99	1.00	1.00	0.00†	0.00†	0.00†
	**NRMSE**	0.08	0.05	0.06	0.03	0.04	0.10	0.15	0.16
F. graminearum + F. verticillioides	**R^2**	0.83	0.96	0.91	0.97	0.97	0.62	0.00	0.79
	**R^{2*}**	0.95	0.99	0.99	0.99	1.00	0.00†	0.00†	0.00†
	**NRMSE**	0.12	0.06	0.08	0.05	0.05	0.12	0.07	0.11
F. graminearum + A. flavus	**R^2**	0.98	1.00	0.94	0.97	0.98	0.99	0.95	0.98
	**R^{2*}**	0.99	1.00	0.99	1.00	1.00	1.00	0.99	1.00
	**NRMSE**	0.05	0.02	0.06	0.05	0.04	0.03	0.05	0.05
F. verticillioides + A. flavus	**R^2**	0.94	0.97	0.88	0.92	0.92	0.97	0.96	0.98
	**R^{2*}**	0.98	0.99	0.99	0.99	0.99	1.00	0.99	1.00
	**NRMSE**	0.07	0.06	0.08	0.07	0.07	0.05	0.05	0.04
F. graminearum + F. verticillioides + A. flavus	**R^2**	0.91	0.96	0.94	0.97	0.98	0.97	0.96	0.96
	**R^{2*}**	0.98	0.99	0.99	0.99	1.00	1.00	0.99	0.99
	**NRMSE**	0.08	0.06	0.07	0.05	0.04	0.05	0.05	0.05

Supplementary Figure 3. Logistic model goodness-of-fit across all 56 treatment–temperature combinations, reported as classical coefficient of determination (R^2), study-specific empirical fit quality score (R^{2*}), and normalized root mean square error (NRMSE). Models were fitted to baseline-corrected ΔOD time series by nonlinear least-squares regression. Values marked with a dagger (†) indicate fits that did not meet the predefined acceptance criteria (ΔODmax ≥ 0.10 and/or R^{2*}< 0.95). These fits were excluded from interpretation of logistic parameters (K, r, t_0) in the main text.

	10 °C	15 °C	20 °C	25 °C	30 °C	35 °C	40 °C	45 °C
A. flavus	e	c	e	e	c	bc	b	a
F. graminearum	bcd	a	a	c	b	d	c	b
F. verticillioides	a	a	b	a	b	d	c	b
F. graminearum + F. verticillioides	bc	a	cd	b	b	d	c	b
F. graminearum + A. flavus	d	b	cd	cde	a	c	b	a
F. verticillioides + A. flavus	ab	a	de	de	c	a	a	a
F. graminearum + F. verticillioides + A. flavus	cd	a	c	cd	a	b	ab	a
**ANOVA p-value**	<0.001	<0.001	<0.001	<0.001	<0.001	<0.001	<0.001	<0.001

Supplementary Figure 4. Statistical comparison of $ΔOD_{max}$ among treatments at each incubation temperature (10–45 °C), based on one-way ANOVA followed by Tukey's HSD test (n = 12 wells per treatment); α = 0.05. Cells report compact letter display within each temperature, treatments sharing at least one letter are not significantly different. Overall one-way ANOVA p-value for each temperature is reported.

Fungus	Co-culture	10 °C	15 °C	20 °C	25 °C	30 °C	35 °C	40 °C	45 °C
A. flavus	Fg	0.49	0.51	0.29	0.19	0.85	-0.16	-0.02	-0.17
	Fv	0.77	0.81	0.08	0.13	-0.03	0.99	0.16	-0.14
	Fg + Fv	0.58	0.78	0.38	0.37	0.68	0.31	0.10	-0.12
F. graminearum	Af	-0.05	-0.10	-0.50	-0.11	0.17	0.32	0.36	0.13
	Fv	0.01	0.05	-0.50	0.29	0.00	-0.07	-0.02	-0.01
	Af + Fv	-0.02	0.01	-0.47	-0.04	0.10	0.50	0.41	0.15
F. verticillioides	Af	-0.02	-0.03	-0.31	-0.62	-0.16	0.67	0.39	0.12
	Fg	-0.05	0.00	-0.24	-0.27	-0.01	-0.03	0.01	-0.01
	Af + Fg	-0.08	-0.04	-0.21	-0.54	0.08	0.45	0.37	0.13

Supplementary Figure 5. Interaction effects on growth performance across temperatures, expressed as ΔNPI (difference in normalized performance index between co-culture and monoculture) for each focal species. Positive values indicate enhanced growth, negative values indicate suppression relative to monoculture at the same temperature. Abbreviations: Af = *A. flavus*, Fg = *F. graminearum*, Fv = *F. verticillioides*.

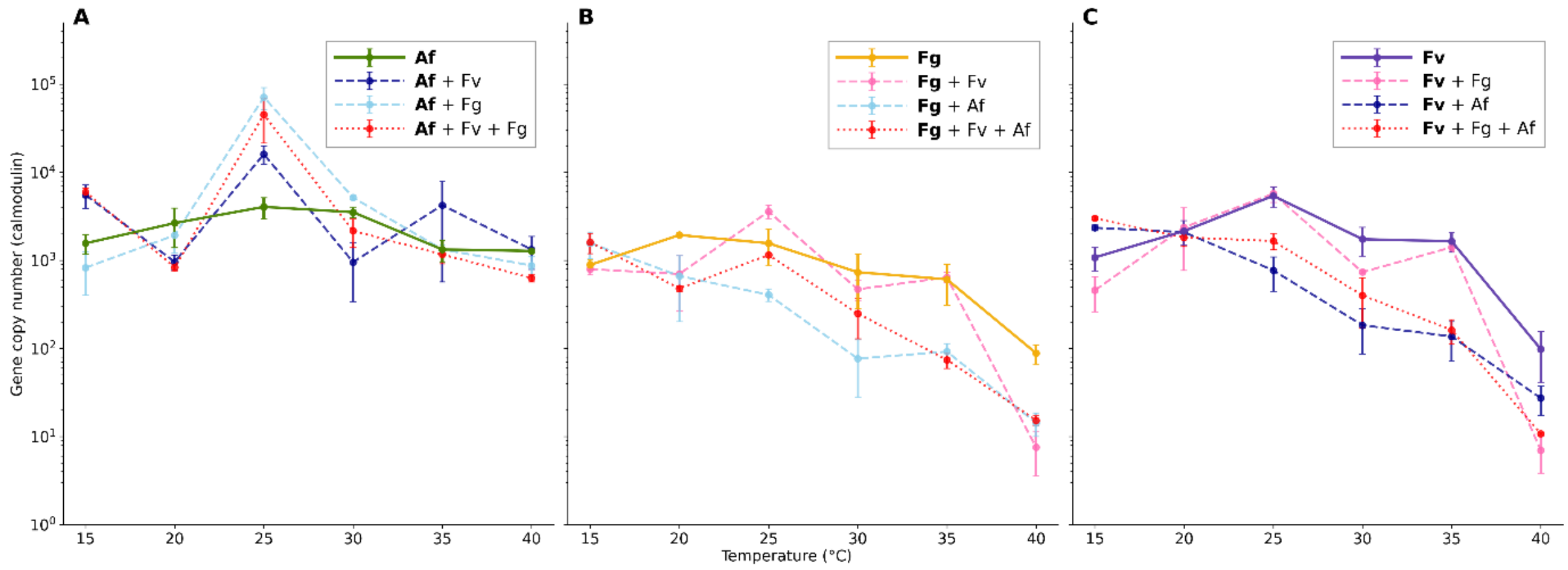

Supplementary Figure 6. Absolute calmodulin gene copy number (GCN) of (A) *A. flavus* (Af), (B) *F. graminearum* (Fg), and (C) *F. verticillioides* (Fv) in monoculture and co-culture across incubation temperatures (15–40 °C), quantified by species-specific qPCR using standard curves. Data are visualized as mean ± SD (n = 3 biological replicates).

Fungus	Co-culture	15 °C		20 °C		25 °C		30 °C		35 °C		40 °C	
		p-value	Sig.	p-value	Sig.	p-value	Sig.	p-value	Sig.	p-value	Sig.	p-value	Sig.
A. flavus	Af + Fv	0.046	*	0.143	ns	0.023	*	0.006	**	0.303	ns	0.883	ns
	Af + Fg	0.092	ns	0.447	ns	0.028	*	0.014	*	0.997	ns	0.082	ns
	Af + Fv + Fg	0.002	**	0.129	ns	0.096	ns	0.073	ns	0.568	ns	<0.001	***
F. graminearum	Fv + Fg	0.367	ns	0.039	*	0.020	*	0.418	ns	0.870	ns	0.020	*
	Fg + Af	0.149	ns	0.041	*	0.100	ns	0.126	ns	0.096	ns	0.024	*
	Fg + Fv + Af	0.086	ns	<0.001	***	0.411	ns	0.198	ns	0.091	ns	0.027	*
F. verticillioides	Fv + Fg	0.057	ns	0.842	ns	0.788	ns	0.110	ns	0.430	ns	0.111	ns
	Fv + Af	0.009	**	0.891	ns	0.025	*	0.048	*	0.022	*	0.163	ns
	Fv + Fg + Af	0.004	**	0.508	ns	0.038	*	0.053	ns	0.024	*	0.119	ns

Supplementary Figure 7. Statistical results for qPCR comparisons of co-culture versus monoculture across species, combinations, and temperatures. Group differences were tested using Welch's two-sample t-test (unequal variances; n = 3 biological replicates per group). Exact p-values are reported together with significance class (Sig.: ns, *, **, *** corresponding to $p < 0.05$, 0.01, and 0.001). Abbreviations: Af = *A. flavus*, Fg = *F. graminearum*, Fv = *F. verticillioides*.
